# Correspondence

**Published:** 1874-09

**Authors:** 


### Correspondence,

PROF. J. TAFT:

*Dear Sir*, According to the reporter of proceedings of the joint sessions of the Mississippi Valley Dental Association and Missouri State Dental Society, as published in the DENTAL REGISTER for June, page 259, in the discussion of "treatment of deciduous teeth" I am made to say: "That if there was any case where the operation of filling the roots was admissible, it certainly was in the case of deciduous teeth," etc. Now I did not say anything of the kind, by reading the above it might easily be inferred that I condemned all root filling in general, while acknowledging its admissibility in deciduous teeth.

What I did say was this: If there was any case *now a days*, in which the operation of *hullihen* was admissible, it certainly was in the case of deciduous teeth, etc. Thus materially altering the sense of the statement.

Will you be good enough to make this correction in the present issue of the REGISTER. F. H. REHWINKEL.

---

ED. DENTAL REGISTER

*Dear Sir:*—An unfailing remedy for the intense pain experienced oftentime after extraction has long been sought for and with a measured degree of success. Chloroform, etc., have long been in use and found to give relief in very many cases, but is not a plesant remedy and if carelessly used is liable to cause injury to the tissues with which it comes in contact.

Recently I have been using Hydrate of Chloral in simple syrup—(a saturated solution) with better success than anything I have used heretofore. It is not unpleasant to use and the relief is complete and immediate, from three to five drops being introduced into the cavity on cotton.

Very respectfully yours,

J. H. WARNER.

